# A framework for ontology-based question answering with application to parasite immunology

**DOI:** 10.1186/s13326-015-0029-x

**Published:** 2015-07-17

**Authors:** Amir H. Asiaee, Todd Minning, Prashant Doshi, Rick L. Tarleton

**Affiliations:** THINC Lab, Department of Computer Science, University of Georgia, Athens, GA USA; Tarleton Research Group, Department of Cellular Biology, University of Georgia, Athens, GA USA

**Keywords:** Chagas, Natural language, Ontology, Parasite data, Question answering

## Abstract

**Background:**

Large quantities of biomedical data are being produced at a rapid pace for a variety of organisms. With ontologies proliferating, data is increasingly being stored using the RDF data model and queried using RDF based querying languages. While existing systems facilitate the querying in various ways, the scientist must map the question in his or her mind to the interface used by the systems. The field of natural language processing has long investigated the challenges of designing natural language based retrieval systems. Recent efforts seek to bring the ability to pose natural language questions to RDF data querying systems while leveraging the associated ontologies. These analyze the input question and extract triples (subject, relationship, object), if possible, mapping them to RDF triples in the data. However, in the biomedical context, relationships between entities are not always explicit in the question and these are often complex involving many intermediate concepts.

**Results:**

We present a new framework, OntoNLQA, for querying RDF data annotated using ontologies which allows posing questions in natural language. OntoNLQA offers five steps in order to answer natural language questions. In comparison to previous systems, OntoNLQA differs in how some of the methods are realized. In particular, it introduces a novel approach for discovering the sophisticated semantic associations that may exist between the key terms of a natural language question, in order to build an intuitive query and retrieve precise answers. We apply this framework to the context of parasite immunology data, leading to a system called AskCuebee that allows parasitologists to pose genomic, proteomic and pathway questions in natural language related to the parasite, *Trypanosoma cruzi*. We separately evaluate the accuracy of each component of OntoNLQA as implemented in AskCuebee and the accuracy of the whole system. AskCuebee answers 68 % of the questions in a corpus of 125 questions, and 60 % of the questions in a new previously unseen corpus. If we allow simple corrections by the scientists, this proportion increases to 92 %.

**Conclusions:**

We introduce a novel framework for question answering and apply it to parasite immunology data. Evaluations of translating the questions to RDF triple queries by combining machine learning, lexical similarity matching with ontology classes, properties and instances for specificity, and discovering associations between them demonstrate that the approach performs well and improves on previous systems. Subsequently, OntoNLQA offers a viable framework for building question answering systems in other biomedical domains.

**Electronic supplementary material:**

The online version of this article (doi:10.1186/s13326-015-0029-x) contains supplementary material, which is available to authorized users.

## Background

New biomedical data is increasingly housed in resource description framework (RDF) triple stores such as Virtuoso [1] and AllegroGraph [2], annotated using rich ontology schemas and queried using an RDF query language called SPARQL [3]. The RDF data model has the advantage of making the relationships between the data items explicit, and provides a straightforward way for annotating data using ontologies. An example of this is the semantic problem solving environment for the immunology of the parasite, *Trypanasoma cruzi (T. cruzi)*, which utilizes an RDF triple store for hosting the parasite’s genomic (microarray), proteomic (transcriptome) and pathway data [4]. The data is annotated using the parasite experiment ontology (PEO) and queried using the open-source Cuebee [5] that provides an interface for facilitating the parasitologist’s formulation of SPARQL queries. Another example is the translational medicine ontology and knowledge base [6], which utilizes the unifying ontology to annotate integrated genomic, proteomic and disease data, along with patient electronic records. The data may be browsed in a RDF triple store.

Simple Web-based forms that allow choosing attributes have been the user interface of choice for traditional biomedical relational databases [7]. To promote ease of querying, systems that utilize ontology-based RDF data have experimented with various interfaces: iSparql [8], NITELIGHT [9] and BioSPARQL [10] facilitate formulating SPARQL queries by allowing the biomedical scientists to browse ontology concepts and pinpoint a subgraph that pertains to the question in his or her mind. GINSENG [11], a guided-input natural language search engine, and Cuebee [5] progressively guide the scientists by suggesting concepts and relationships that decompose the question into a RDF triple based query, which is then internally translated into SPARQL. The triples are in the form of subject → relationship → object where subject and object represent ontology concepts. As Asiaee et al. [12] notes, such guidance is tightly coupled to the particular ontology structure, and query formulation requires comfort with the structure otherwise the final query is unintuitive to the user.

In this article, we introduce a novel framework, OntoNLQA, for querying RDF data annotated using ontologies. The specific contributions of this framework are:

1. It allows posing queries as natural language questions thereby requiring minimal translation between the question in user’s mind and the computer query.
2. We present a new approach for answering natural language questions on structured data that combines machine learning with semantic computing: use of existing ontologies, their structure and annotated data, and triple-based queries.
3. OntoNLQA is applied in the context of parasite immunology. The resulting system called AskCuebee allows parasitologists to pose genomic, proteomic and pathway questions in natural language related to the parasite, *T. cruzi*, for the first time.
4. AskCuebee automatically answers 68 % of a corpus of 125 questions in 5-fold cross-validation, and 60 % of the questions in a previously unseen corpus. This latter proportion increases to 82.5 % if we allow simple corrections by the user to the output of some of the components.

OntoNLQA is significant due to two reasons: First, it improves on the disadvantages of existing biomedical data retrieval systems. In a systematic evaluation, Asiaee et al. [12] demonstrate the benefits and limitations of existing ontology-driven query formulation systems. A major limitation is that scientists using these systems require an understanding of the ontology structure in order to quickly formulate queries. For example, queries may require using intermediate concepts in the ontology when there is no direct relationship between the concepts that scientists have in mind.

**Fig. 1:**
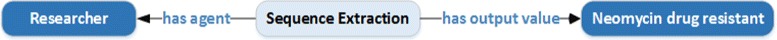
In order to formulate a query for the question above, the scientist needs to relate the two concepts, *researcher* and *Neomycin drug resistance*, using the intermediate concept in the ontology, *sequence extraction*, that connects the two. Realizing how these are related requires an understanding of the ontology design and its structure

To illustrate, consider this question in the context of *T. cruzi* immunology using the parasite experiment ontology (PEO) [13]: *Which researchers study Neomycin drug resistance?* PEO formalizes the experimentation processes in parasite research. Figure 1 illustrates the connection between the two concepts *researcher* and *Neomycin drug resistance* in PEO. Notice that “study” corresponds to a path that includes two ontology properties, *has agent* and *has output value*, and an intermediate ontology class, *sequence extraction*, which is not stated in the question. Because questions may not explicitly state how the target concepts are related, the scientist’s RDF query is tied down to the structure of the ontology and this problem exaggerates when multiple intermediate concepts are needed.

Second, and the more important motivation derives from the fact that a capability to pose questions in plain language is a natural way of obtaining answers. It involves minimal effort expended toward translating the question in the scientist’s mind to a query acceptable to the system, which includes the effort involved in acquainting with the query interface. In our informal discussions with biomedical scientists, this capability was consistently identified as the one that is most preferred.

OntoNLQA seeks to automatically translate a question into RDF triples, and build a SPARQL query to retrieve the answers from data stored in the RDF data model. Toward this, the framework utilizes a design comprised of *five components* working in a sequence: The first two identify important entities in the scientist’s question. These are constituent words that are nouns and verbs, and relate to the concepts and relationships in the domain. Accuracy is important here because words erroneously deemed important get carried forward through all the components. The third component matches the entities identified previously to classes and properties of the ontology. The last two components receive a set of ontology classes and properties, and find semantic associations between the entities. These associations could be multiple paths comprised of classes and properties represented as sequences of RDF triples, which are translated into SPARQL to query the RDF data.

OntoNLQA is not specific to a domain with multiple strategies and methods possible to realize each component. We instantiate this framework in the context of parasite immunology, and develop a system called AskCuebee that allows parasitologists to pose genomic, proteomic and pathway questions in natural language related to the parasite, *T. cruzi*. A significant amount of data including internal laboratory data sets, KEGG pathway data, and genomic data on orthologs such as *Leishmania major* and *Trypanosoma brucei* from TriTrypDB [7] is available in a RDF store for querying. The data is annotated using PEO.

We evaluate the accuracy of each component of OntoNLQA as implemented in AskCuebee, and the accuracy of the whole system.

AskCuebee has been deployed in the Tarleton lab for use in their day-to-day research and replaces a previous traditional relational database system^1^.

While the field of natural language processing has long investigated the challenges of designing systems that respond to questions in natural language [14–18], these do not make use of ontologies or the RDF data model. Few recent ontology-based retrieval systems [19, 20] allow queries as natural-language questions and seek to extract subject → predicate → object triples directly from the input question using pattern matching. However, a significant limitation is that the extracted triples may not be present as is in the ontology because the scientist’s question may not be cognizant of the ontology’s structure. Furthermore, as we illustrated previously, entities in the question may not be directly related motivating sophisticated ways of connecting them to form an intuitive query. Consequently, a large subset of the questions are challenging to answer automatically, thereby necessitating user involvement to refine the triples. For example, Aqualog [19] could not answer 42 % of the questions in its corpus automatically resorting to manual intervention for these questions. A small subset of the systems [11, 21] refine the question in real-time – as it is being typed – by suggesting concepts and relationships from the ontology to the scientist. These occupy a middle ground between those that truly allow questions in natural language and those in which queries are RDF triples.

Our driving biomedical domain pertains to the immunology and pathogenesis of the parasite *T. cruzi* infection, which causes the Chagas disease. This disease was recently labeled the “new HIV/AIDS of the Americas” [22]. About 7 million people, mostly in Latin America, are infected with this disease. Data available for querying by AskCuebee was collected in order to study how immune control and maintenance occurred during a *T. cruzi* infection and how it managed to avoid immune clearance. Data on DNA cloning steps for gene knockout studies, on generation of gene knockout strains, whole-genome transcript abundances for the four life-cycle stages of *T. cruzi*, orthologous genes in related organisms and protein identifications based on peptide spectra are all included as RDF data.

### Article outline

Next, in the Methods section, we describe the design of OntoNLQA, discuss the details of each component and how each component is utilized in AskCuebee in the context of the parasite, *T. cruzi*, immunology research. We report on the results of evaluating the methods employed in AskCuebee as well as demonstrate the performance of each component and the performance of the whole system in the Results and discussion section. We also discuss the contributions and limitations of our framework based on our evaluation results in this section. We present a comprehensive review of related work with a focus on ontology-based retrieval systems in Related Works. We conclude the article by summarizing our approach, main findings and providing thoughts on future directions in the Conclusion section.

## Methods

OntoNLQA presents a new approach for answering questions posed in natural language to RDF data annotated using an ontology. We begin by providing an overview of the framework followed by details on each component and how it is applied in the context of a driving biological domain, as well as its evaluation. As we discuss below, multiple alternatives present themselves for realizing each component of the framework, and we discuss their benefits and limitations.

### Overview of OntoNLQA

Briefly, our approach in OntoNLQA is to identify the important entities present in the question, which are then found in the ontology and semantic associations between the entities in the ontology are discovered. This approach encounters three main challenges:

1. OntoNLQA needs to parse the question and identify the important entities;
2. It must find the ontology classes, properties and instances (data) in the ontology(ies) that correspond to the identified entities; and
3. Find semantic associations involving the ontology classes and properties, which need not be on a single directed path. Express these in the form of RDF triples that are translated into a computational query for the RDF data.

While these challenges are common to some of the previous ontology-based natural language systems, OntoNLQA differs in its approach toward addressing them.

**Fig. 2:**
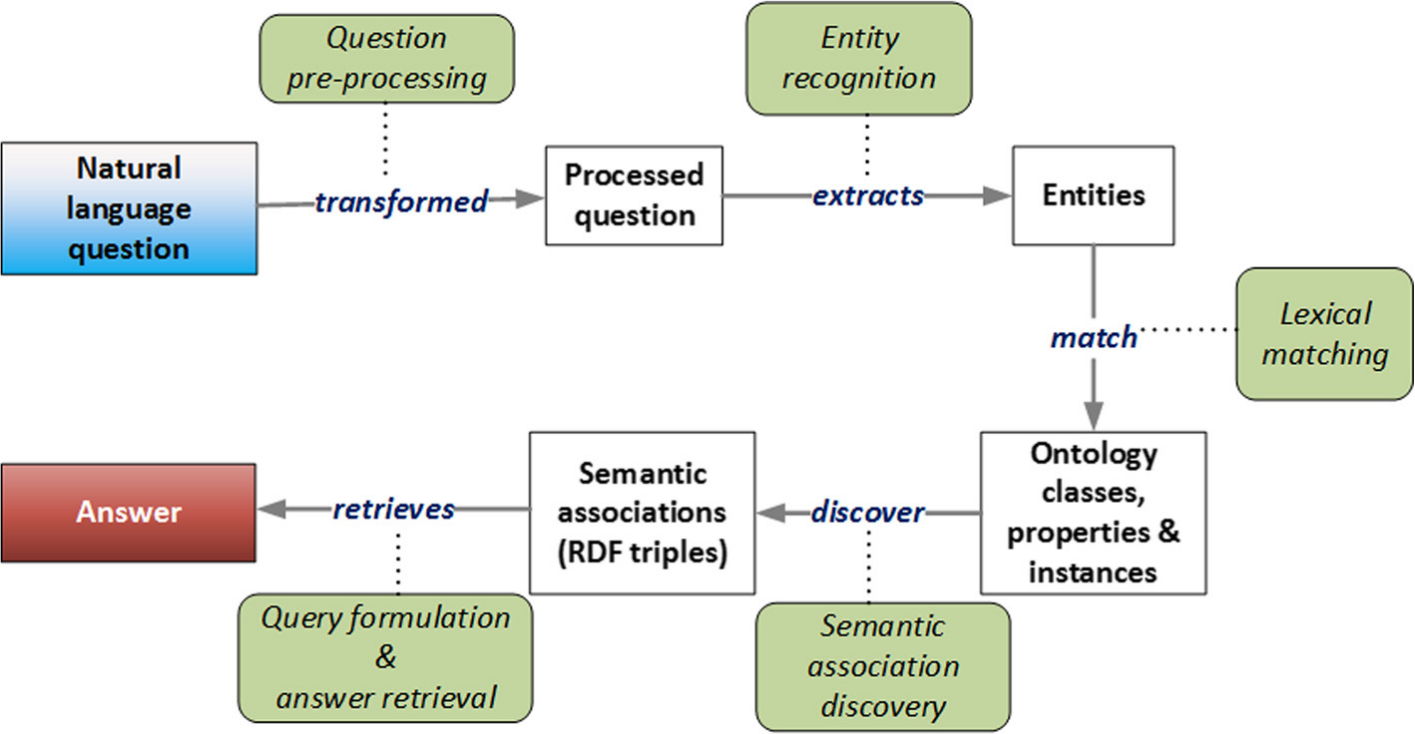
An illustration of the flow of data in OntoNLQA emphasizing the operation performed on the data at each step. Dotted lines show the operation on the data. For example, *lexical matching* gives the *ontology classes*, properties and *instances* similar to the *extracted entities*. The direction of the arrows denotes the direction of flow of the data

These challenges suggest a pipeline of operations on the data beginning with the question in natural language, as illustrated in Fig. 2. On receiving a question in natural language, OntoNLQA performs *linguistic pre-processing* of the question during which punctuation symbols, quotation marks, parenthesis and any other character in the question generally deemed to be irrelevant to extracting the important information, are filtered out. This results in a *processed question*. Words and phrases relevant to the domain and of import to understanding the question are deemed as important *entities* and extracted from the processed question by utilizing entity recognition techniques. These entities are then found in the ontology using lexical matching. Ontology classes matched to the entities form the *end points* of any semantic associations that are additionally constrained to include matched ontology properties, if any. These associations are represented as a sequence of *RDF triples*, which are then transformed into SPARQL queries that retrieve the answer.

**Fig. 3:**
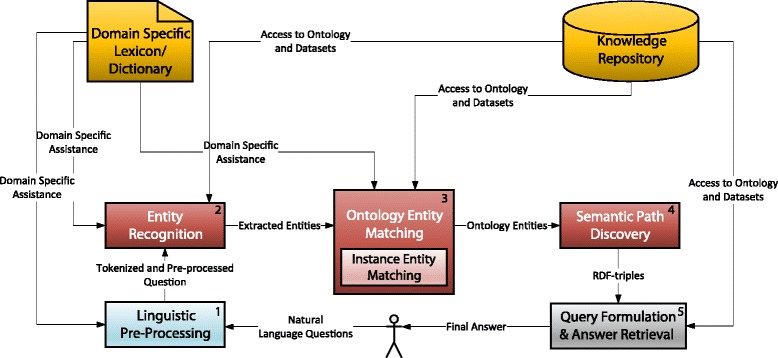
The design of OntoNLQA involving five general components that operate on the scientist’s question to eventually obtain the answer

Operations on the data in Fig. 2 are performed by the components of the system. Subsequently, OntoNLQA is composed of *five* components as we show in Fig. 3. The first two components, which include linguistic pre-processing (box annotated 1 in Fig. 3) and entity recognition (box annotated 2) address the first challenge, which is similar to the well-known problem of named entity recognition [23]. Our primary goal in extracting entities is to match them with their corresponding ontology elements. Therefore, the labels in our context is a set of ontology classes and properties. A third component (box annotated 3) matches each extracted entity from the previous component to a specific ontology class, property or instance.

This component addresses the second challenge of finding corresponding ontology elements for the identified entities.

The final two components handle the challenge of finding relationships between the ontology elements, representing them as RDF triples, and translating these into a computational query. Semantic association discovery is nontrivial when more than two ontology elements need to be related (box annotated 4). Discovered semantic associations may be represented as RDF triples. These are used in generating a computational query for the RDF data by the query formulation and answer retrieval component (box annotated 5).

### Components of OntoNLQA and their Implementation in AskCuebee

In this subsection, we describe the components of the framework in detail. For each, we discuss various methods for realizing the component’s functionality, which may be beneficial in different contexts, and its utilization in AskCuebee.

**Fig. 4:**
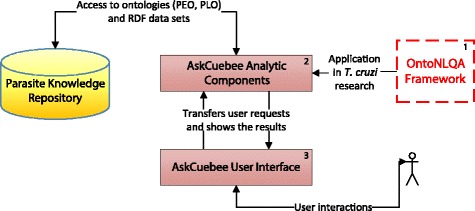
OntoNLQA is a general framework and its realization in the context of our driving biological problem is called AskCuebee

We apply OntoNLQA to the context of *T. cruzi* parasite immunology data as illustrated in Fig. 4. We call this application, AskCuebee (boxes annotated 2 and 3), which is assisting parasitologists at the Center for Tropical and Emerging Diseases at the University of Georgia, and their collaborators worldwide. The parasite, *T. cruzi*, is the agent of Chagas disease in humans. This disease is prevalent throughout Latin America and is often fatal.

#### Linguistic pre-processing

All questions undergo linguistic pre-processing to filter constituents that are not key toward a computational understanding of the question. This pre-processing – commonly utilized in many question-answering systems – is generally known to improve the accuracy of detecting important entities. The pre-processing starts with tokenization: breaking down the string of characters into words, phrases, symbols, or other meaningful elements. This is followed by removing stop words such as the definite articles, “to”, “was”, and many others. Standard lists of stop words are available [24]. In addition, punctuation symbols are removed, abbreviations are expanded, and comparative relationships in words are identfied using grammar dependencies [25] and parts of speech tagging [26], and replaced by their mathematical symbols; for example, “greater than 1” is replaced by “ >1”.

The accuracy of linguistic pre-processing may be enhanced by using domain-specific lexicons or dictionaries such as UMLS or MeSH, if these are relevant, though its use should be considered carefully due to the concomitant increase in run time [27]. Much of the previously mentioned functionality for pre-processing is available in free computer applications such as Stanford CoreNLP [28], LingPipe [29] and OpenNLP [30].

##### StanfordCoreNLP in AskCuebee

Each question posed by the user is viewed by the system as a string of characters. Therefore, common operations such as tokenization, extracting the roots of words (stemming), and removing the punctuation symbols are essential. AskCuebee performs these using the *standard* operations found in the Stanford CoreNLP library [28]. Furthermore, consider the following two example questions:

1. *Show me the 3 prime forward sequences for all genes in metacyclic stage with log2 ratio higher than 1 and standard deviation below 0.5.*
2. *Which protein group numbers have spectral values between 40 and 50?*

In question (1), notice that while there are three numbers mentioned, two of these are involved in comparative relationships, 1 and 0.5. Thus, the comparative relationships we seek to identify are *log2 ratio > 1* and *standard deviation < 0.5*. In question (2), the comparative relationships are more complex as two relationships are combined into one using a conjunction. Therefore, we seek to extract two relationships, *spectral values > 40* and *spectral values < 50*. These questions illustrate that we additionally need conversions between numbers and text, and extraction of comparative relationships. Both these require complex operations that include part-of-speech tagging such as detecting the nouns, verbs and identifying grammar dependencies, which are provided by Stanford CoreNLP. In addition, we detect abbreviations from a list that we maintain.

We introduce a simple method that uses dependency grammar to detect the majority of the comparative relationships. The first step is to detect the comparative phrases in the question and transform them into distinct patterns. For example, *standard deviation below 0.5* from question (1) is transformed to *standard deviation *less than* 0.5* and *spectral values *between* 40 and 50* from question (2) is converted to *spectral values *greater than* 40 *and* spectral values *less than* 50*. Next step converts the distinct patterns into a computational form by identifying the operands (*standard deviation* and 0.5) and operators (*less than*). Again, a dependency grammar is combined with part-of-speech tagging to create rules for detecting operands and operators.

#### Entity recognition

Given the processed question, this component in the framework is tasked with identifying and labeling entities that are relevant to obtaining the answer. Several approaches may be used toward entity recognition.

These include supervised learning – a branch of machine learning – that utilizes statistical models for the task. A classifier is trained using a large corpus of data records, each of which is labeled with the target entity names. Entities in new data records are then identified and labeled by the classifiers. Potential classifiers include the hidden Markov model [31–34], maximum-entropy Markov model [35, 36], support vector machines [37], and conditional random fields [38], all of which have been utilized for entity recognition. Among these, conditional random fields have distinguished themselves with their comparatively more accurate performance [39–41].

Supervised learning usually requires a large training corpus to learn a classifier that performs well. In the absence of large data sets, the alternative method of semi-supervised learning uses a small collection of data to train an initial classifier, which is then used to label new and previously unseen samples of data. These labeled data are subsequently utilized to retrain the classifier. A common technique for semi-supervised learning is bootstrapping, which requires a small set of seed supervised data for the initial learning [42].

Other approaches not based on machine learning rely on dictionaries and rules. A simple approach is to locate lexically similar dictionary terms for each potential entity in the question [43–46]. The approaches differ in how they search the dictionary with some using BLAST [47], and the data sets that constitute the dictionary. For example, Krauthammer et al. [43] utilizes GeneBank as the dictionary. Alternately, general rules in the form of string patterns may be available. If a rule is satisfied by a term and its context in the question, the corresponding label is used to annotate the term [48–51].

Between the different approaches for entity recognition, machine learning methods are currently receiving increased attention in general [52, 53]. Regardless of semi- or fully-supervised methods, we need a set of labels for entity recognition. Presence of a domain ontology provides a natural source for these labels. In this regard, an important consideration is the number of labels that are needed, which is often proportional to the size of the data set. A large data set may permit better discrimination and therefore more labels. On the other hand, a smaller data set necessitates fewer labels. In this case, we may select ontology classes and properties that appear at a higher hierarchical level in the ontology graph. Let $\mathcal {C}_{O}$ denote this set from ontology, *O*. Such labels tend to be general, and each is useful toward annotating several terms in the question.

##### Conditional random fields for entity recognition in AskCuebee

Dictionary-based methods require domain-specific dictionaries. While substantial overarching dictionaries for biomedicine such as UMLS and MeSH are indeed available for use, these are not designed to be specific to any particular organism. Biomedical ontologies, if available, serve to provide another source of dictionary terms usually specific to a domain. In addition to finding a dictionary relevant to the domain of interest, a limitation of this approach is that dictionary look up could get expensive if the dictionary is very large and unindexed. On the other hand, machine-learning based supervised classification may need large training data in order to achieve reasonable performance.

Among supervised learning methods, conditional random fields (CRF) [38] demonstrate superior performance for biomedical entity recognition. For example, CRFs were utilized by the best performing system on the i2b2 medical concept extraction task [41], by highly ranked systems on BioCreAtIve gene mention recognition tasks [39, 40] (9 of 19 highest ranked systems use CRFs) and on JNLPBA bioentity recognition task [54]. This motivates considering CRFs in AskCuebee as well. We briefly review CRFs in Appendix Appendix A.

AskCuebee employs a linear-chain CRF and a popular quasi-Newton method called limited memory Broyden-Fletcher-Goldfarb-Shanno [55] for optimizing parameters. The parameters are the feature weights, *λ*_*j*_, in a CRF.

Critical to the performance of CRFs is finding a set of feature functions. The simplest features of a natural language question are the word tokens themselves. In addition, AskCuebee uses four different types of features for training CRFs: orthographic, word shape, dictionary and contextual features:

- *Orthographic features*: Biomedical entities often share orthographic characteristics. These consist of capitalized letters, include digits and possibly some special characters as well. Thus, such features are not only useful in detecting various types of biomedical entities but are easily implemented using patterns or regular expressions. Appendix Appendix A includes a list of the orthographic features utilized in AskCuebee.
- *Word shape*: Words annotated with the same entity label may have the same shape. For example, a type of abbreviation may not have numerical digits and gene IDs are a combination of digits and letters.
- *Contextual features*: These features take into account the properties of preceding and following tokens for a current token in order to determine the target label.
- *Dictionary features*: For each noun or verb phrase in the input question we calculate their similarity scores with all ontology elements. If the highest similarity score is higher than a threshold (for instance, 0.6), we find the upper-level class or property of that specific ontology element that is a training label. Then, we activate a dictionary feature for the identified training label. This feature is useful when the target entities belong to more than one label.

#### Ontology element matching

Entity labels are ontology classes and properties, which could be general and appear at the higher levels of the ontology hierarchy. However, the RDF data annotated by the ontology is often linked to more specific classes and properties. Consequently, we may search the ontology for more specific matches with the recognized entities in the questions. If an entity, *e*, is associated with a label, $c \in \mathcal {C}_{O}$, where $\mathcal {C}_{O}$ is the set of all classes and properties in ontology, *O*, then, let $\mathcal {S}_{c}$ be the set of subclasses and properties in the ontology hierarchy rooted at *c*. *Labeling the entity with c allows us to limit our search for a more specific match to the elements of*$\mathcal {S}_{c}$. Importantly, this reduces the computational expense when the whole ontology may be very large as is often the case with biomedical ontologies.

A suitable approach for the matching is to use text similarity measures to calculate the degree of similarity between an entity and a specific ontology class or property. A similarity measure scores the degree of similarity between two text phrases by viewing them as sequences of characters. Common measures that are utilized include:

- *ISUB similarity* [56] designed for aligning ontologies [57]. This method identifies the longest common substring and records its length. It removes this substring and searches for the next longest substring until no common substring is identified. The sum of the common substrings scaled with the length of the original strings is the commonality in the two strings. ISUB subtracts this commonality from the difference of the two strings. The difference is based on the length of the unmatched substrings.
- *Levenshtein-based similarity (also known as Needleman & Wunsch)* [58] uses the Levenshtein distance [59] to determine the similarity of two sequences of characters. It calculates the best alignment between two sequences of characters as the fewest number of mutations necessary to build one sequence from the other.
- *Smith and Waterman based similarity* [60] looks for longest common substrings between two phrases, and based on that produces the degree of similarity. This measure is similar to Needleman-Wunsch, and is commonly used in BLAST for aligning genome and protein sequences.
- *Cosine-based similarity* [61] is a widely reported measure for similarity between two vectors. This measure models phrases as vectors of characters and calculates the cosine between the two vectors. This provides a score that is interpreted as the degree of similarity between two chunks of texts.
- *Jaccard-based similarity* [62] calculates the degree of similarity of two phrases by calculating the size of the set of intersection of the terms in the two phrases compared to the size of the set of union of the terms.

No particular measure in the above list dominates any other measure in performance. Subsequently, we may evaluate all of them for use in domain-specific systems such as AskCuebee. Classes and properties in $\mathcal {S}_{c}$ that match sufficiently well with the entity, *e*, become a part of the candidate list. Based on the cardinality of the candidate list, three situations arise as discussed below:

*Case (1)*: In the straightforward case where the candidate list has only one member, the matched subclass or property is retained.

*Case (2)*: If the candidate list has multiple members, we need to retain one among them. Here, we may consider the *context*: the other entities identified in the question and how each candidate relates with the ontology classes and properties that label the other entities. For example, we may rank order the candidates based on how many direct paths each has with the other labels found in the ontology. We may retain the candidate with the most paths, which is indicative of contextual consistency.

*Case (3)*: Finally, the candidate list could be empty. Because none of the ontology subclasses or properties were a close lexical match, our next step is to identify a match in the RDF data. We may lookup the *rdfs:type* of the matched instances in the data set to obtain the corresponding ontology classes or properties. If multiple such classes obtain, the candidate list has multiple members requiring the approach in case (2) above to retain one.

#### Semantic association discovery

Specific ontology classes and properties that label the identified entities in the question now need to be related to each other. Two ontology elements have a semantic binary relationship if a directed or undirected path connects them in the ontology graph. However, scientists’ questions often include multiple entities. OntoNLQA differs from previous systems in how it handles this situation. We must find an n-ary semantic relationship between all of them. While pairwise binary relationships may be found between each pair of labels, these paths must be linked with each other.

An approach to relating them is to find the *lowest common ancestor (LCA)*. This is the ontology class that is the ancestor of each entity label. An ancestor is any class that lies on the path from the root of the ontology to the label class. If there are multiple such common ancestors, we pick the one that is most specific and is therefore lowest in the hierarchy. This ancestor would coincide with Resnik’s most informative common ancestor [63] if attention is limited to just the subclass taxonomy of the ontology. However, the latter requires finding the probabilities of ontology classes typically using term frequencies in domain texts. Furthermore, the LCA may be different when named properties in an ontology are considered.

**Fig. 5:**
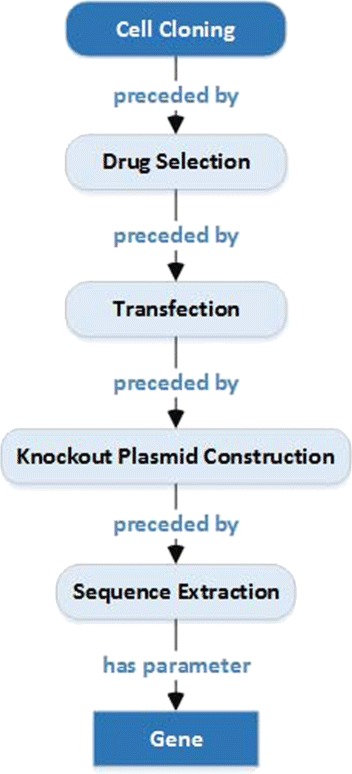
The semantic path between the ontology concepts *Cell Cloning* and *Gene ID Tc00.1047053509463.30*. The lowest common ancestor is *Cell Cloning*

An an illustration, consider Fig. 5 which shows the semantic relationship between labels *Cell Cloning* and *Gene* that appear in PEO. The binary relationship between these two labels is a direct path in PEO. In this path, there are several intermediate ontology concepts such as *drug selection* and *transfection* (marked differently), which are a part of the relationship. Of course, the length of such paths depends on the design and structure of the particular ontology. As there is one pair only in this example, a single path is sufficient to obtain the semantic association between the two labels.

**Fig. 6:**
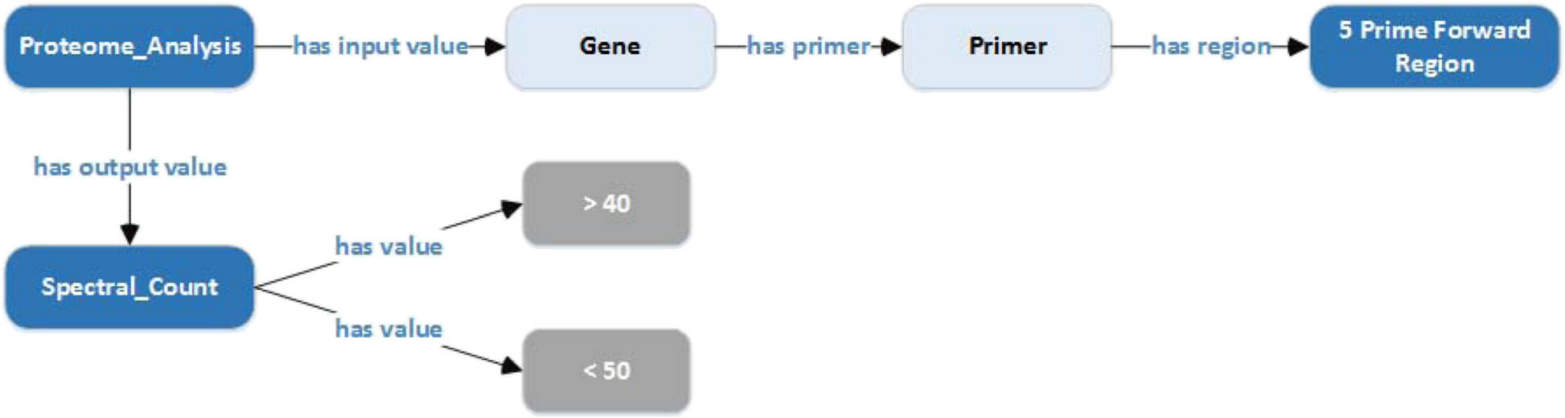
This graph shows the semantic paths between the ontology concepts, *five prime forward regions*, *proteome analysis*, *spectral value > 40* and *spectral value < 50*. The common node between all is *proteome analysis*, which forms the LCA

**Fig. 7:**
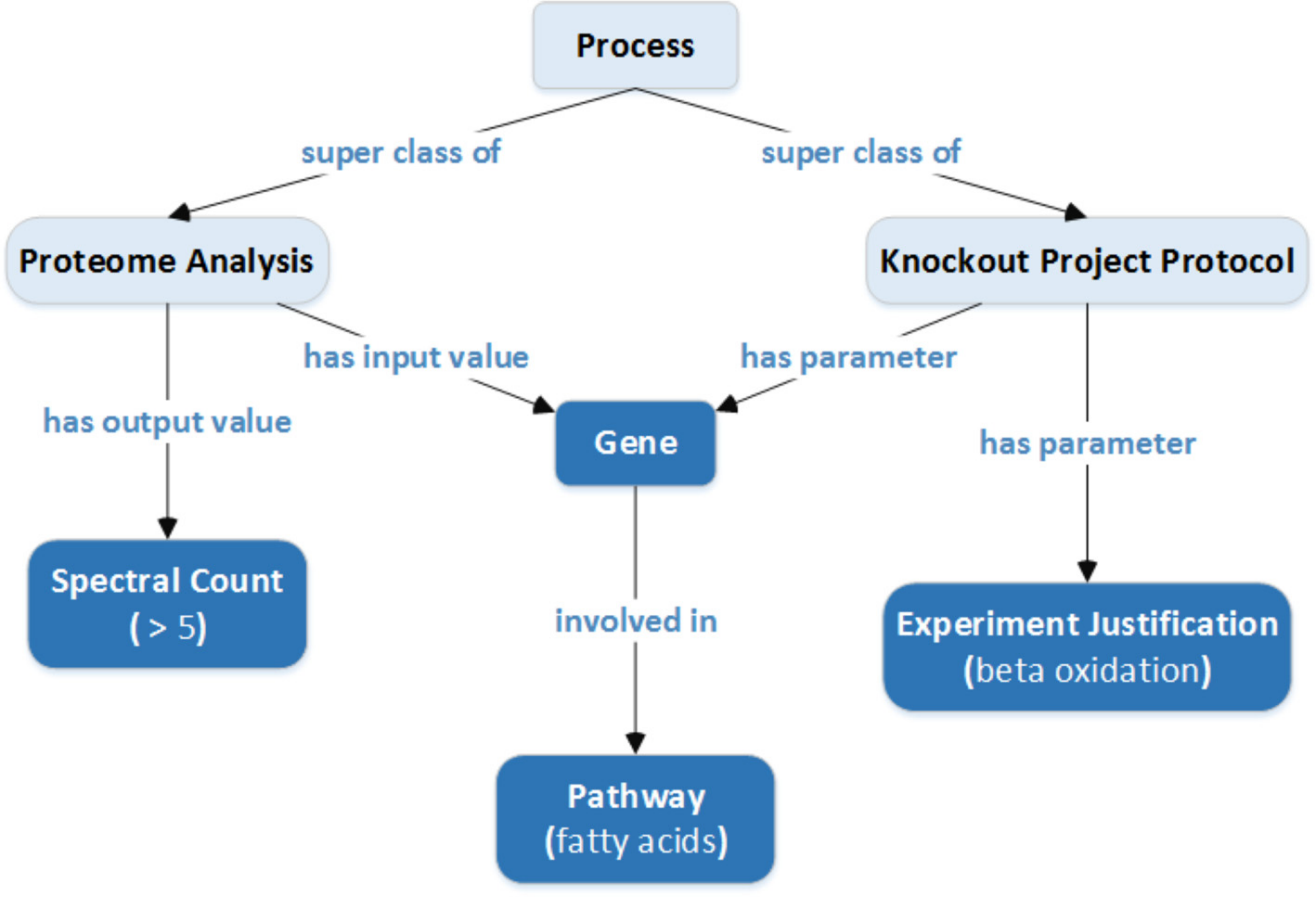
The lowest common ancestor in this example, process, is not contained in the pairwise paths. Rather, it requires tracing paths to the root from each entity label

The graphs in Figs. 6 and 7 consider examples from questions containing more than two identified entities, resulting in more complex semantic associations between the ontology elements. In Fig. 6, we are looking for semantic relationships between *five prime forward region*, *spectral count* and *proteome analysis* concepts, which were chosen as entity labels. This n-ary relationship may not be a single path between the elements. However, there are pairwise paths between each pair of the ontology elements. Notice that *proteome analysis* is present on all these paths and is the LCA.

As a third example, consider the ontology classes, *gene*, *spectral count*, *pathway*, and *experiment justification* in Fig. 7. It is straightforward to find the pairwise paths between the classes in the ontology subgraph. However, unlike the previous example, none of these include a common ancestor. As we show in Fig. 7, the LCA, *process*, is an intermediate concept and does not belong to the set of labels for the entities in the question.

In order to find semantic associations between multiple ontology classes or properties, we discuss two methods below:

##### Method 1: Semantic association discovery based on the LCA

Notice that the presence of an LCA for the matched classes or properties in an ontology provides a way to obtain the semantic association between them. From the LCA, we may obtain the shortest path that connects the LCA to each ontology class while including any identified property. Consequently, we obtain multiple paths each of which has the LCA at one end.

**Fig. 8:**
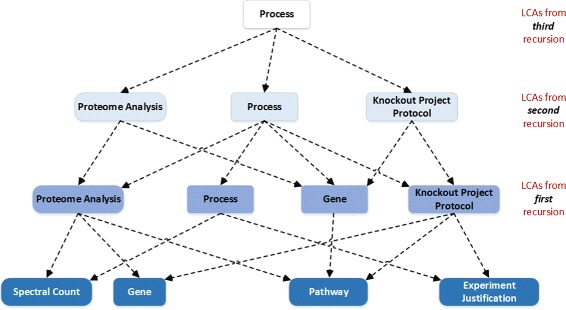
The recursive algorithm finds a single LCA for the ontology entities in Fig. 7. In the first recursion, the algorithm finds LCAs between every pairs for *spectral count*, *gene*, *pathway*, and *experiment justification* nodes. In the second and third recursions, the algorithm finds the LCAs of results of previous recursion until a single node remains, which is *process*

This motivates finding an efficient way to compute the LCA. In Appendix Appendix B, we discuss an offline approach that precomputes the LCA for each pair of classes in the ontology at hand and is currently the fastest. We may then simply look up the LCA table to find all LCAs for every ontology entity pair. This process continues recursively until we identify a single LCA for all of the entity labels. Figure 8 illustrates this recursive algorithm. Note that this recursive procedure iterates over all LCAs for every pair until one of them leads to the final solution. If the algorithm fails to find any LCA for the entities, it concludes that there is no semantic association between the ontology classes. In order to find the shortest path from the LCA to each ontology class in the set of entity labels, we may use bidirectional search [64] to speed up the path finding.

##### Method 2: Semantic association discovery based on path finding

An alternative approach for finding semantic associations is based on path queries. For example, SPARQL 1.1 provides facilities to find a path between two elements in RDF data. We may use these path-finding queries to find the semantic paths between multiple ontology classes and properties. We present a simple method that includes finding all the paths between the ontology elements and selecting a common node among these paths. Specifically,

- We begin by finding pairwise paths: these are paths between every pair of ontology elements in the set of labels. We sort them based on their length in ascending order.
- Note that multiple paths may exist between a pair of ontology elements. We create a set, {*allPairwisePaths*}, that contains sets of all the pairwise paths between every pair of the elements.
- In the next step, a Cartesian product of the sets in *allPairwisePaths* is obtained. Each member of the product set is itself a set of pairwise paths between all the ontology elements.
- For each member of the product set, we identify an ontology class that is common to all the paths, if available, and store these common classes in a set, *CommonNodes*.
- Finally, this approach selects a class in the set, CommonNodes, that has the shortest paths to the ontology elements.

Both the above methods result in semantic paths, which are then converted into sequences of RDF triples in a straightforward way.

##### Association discovery in AskCuebee

In order to discover the associations between the matched PEO classes and properties, OntoNLQA suggests either precomputing the LCA or running path queries between each pair of matched ontology elements and finding their intersection. While the former has an offline step of precomputing the LCA between all pairs of classes in the ontology, the latter is fully online. We evaluate the two approaches and select one for inclusion in AskCuebee.

#### Query formulation and answer retrieval

The final component of OntoNLQA translates RDF triples into a computational query in the language of SPARQL. This translation is straightforward because the RDF triples directly represent SPARQL graph patterns.

If the RDF triple sequences constituting the semantic paths need to be displayed, we may utilize any modality including simply showing the sequences or marking them on the ontology graph and displaying the subgraph. As an example, we may utilize the display of RDF triple sequences by a system such as Cuebee [5].

The SPARQL query is then sent to any query endpoint such as OpenLink Virtuoso [1], OpenRDF Sesame [65] or AllegroGraph [2], all which allow storing large amounts of annotated RDF data and query it using SPARQL. The answers may be displayed to end users in a tabular or any other visual format depending on the context and scientist preferences.

### AskCuebee workflow

**Fig. 9:**
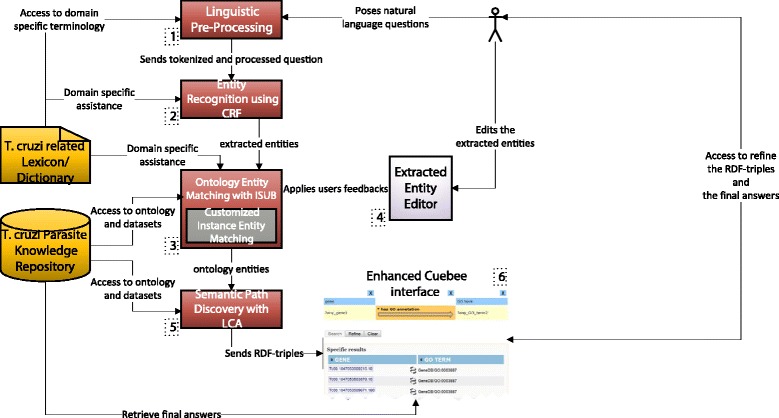
The workflow of AskCuebee, which implements OntoNLQA in the context of *T. cruzi* immunology research. Specific methods are chosen after evaluating the alternatives

AskCuebee implements various components in a workflow that is visualized in Fig. 9. We briefly summarize the internal workflow and provide details below. Linguistic pre-processing of the scientist’s question in natural language (step labeled 1) is performed using a set of standard operations implemented in the Stanford CoreNLP [28] software library. Entities in the processed question are identified and labeled using a machine learning classifier: the conditional random field (step labeled 2). The labels are further refined by matching the entities with ontology classes or properties using a lexical matching algorithm called ISUB (step labeled 3). The scientist may edit the recognized entities and labels for accuracy, and the lexical matching is performed again (step labeled 4). Semantic associations between the specific labels are obtained by finding the shortest paths from the LCA to the ontology elements (step labeled 5). The semantic paths are then passed to an enhanced version of Cuebee [4], which transforms the RDF triples into computational SPARQL queries and retrieves the answers (labeled 6).

### AskCuebee’s user interface

**Fig. 10:**
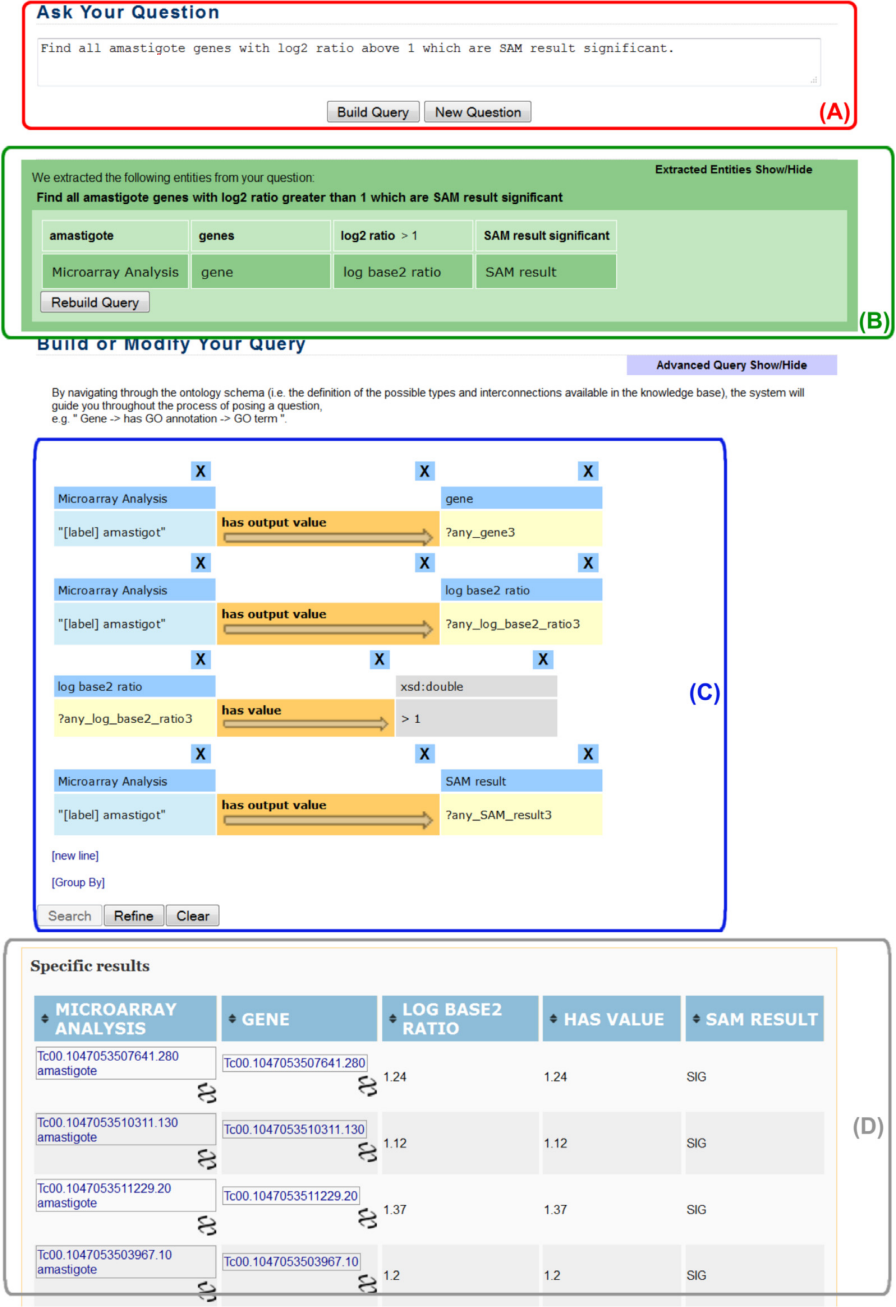
A snapshot of AskCuebee in action: answering a question relevant to *T. cruzi* research. There are four sections in the interface each of which represents the different components of AskCuebee. Section (A) contains a large textbox for the scientist to enter her question and ask the system to create a corresponding query and retrieve the answers by pressing the *Build Query* button. Section (B) is optionally shown and displays the recognized entities in the question, allows the scientist to modify the recognized entities, and search for new labels in the ontology. Figure 11 shows further details of this section. Section (C) shows the sequence of RDF triples that represent the question. This section is integrated with an enhanced version of the existing system, Cuebee, and uses its display and design. Section (D) similar to section (C) is the result of integration with Cuebee and shows the final answers retrieved from the RDF data sets

In Fig. 10, we show a snapshot of AskCuebee’s interface for the user. The scientist may enter her question in its original, natural language form, in section (A) of Fig. 10 followed by pressing the *Build Query* button to send the question to the system. AskCuebee processes the question over a sequence of steps, and the intermediate output from some of the components is displayed to the user in an intuitive manner.

**Fig. 11:**
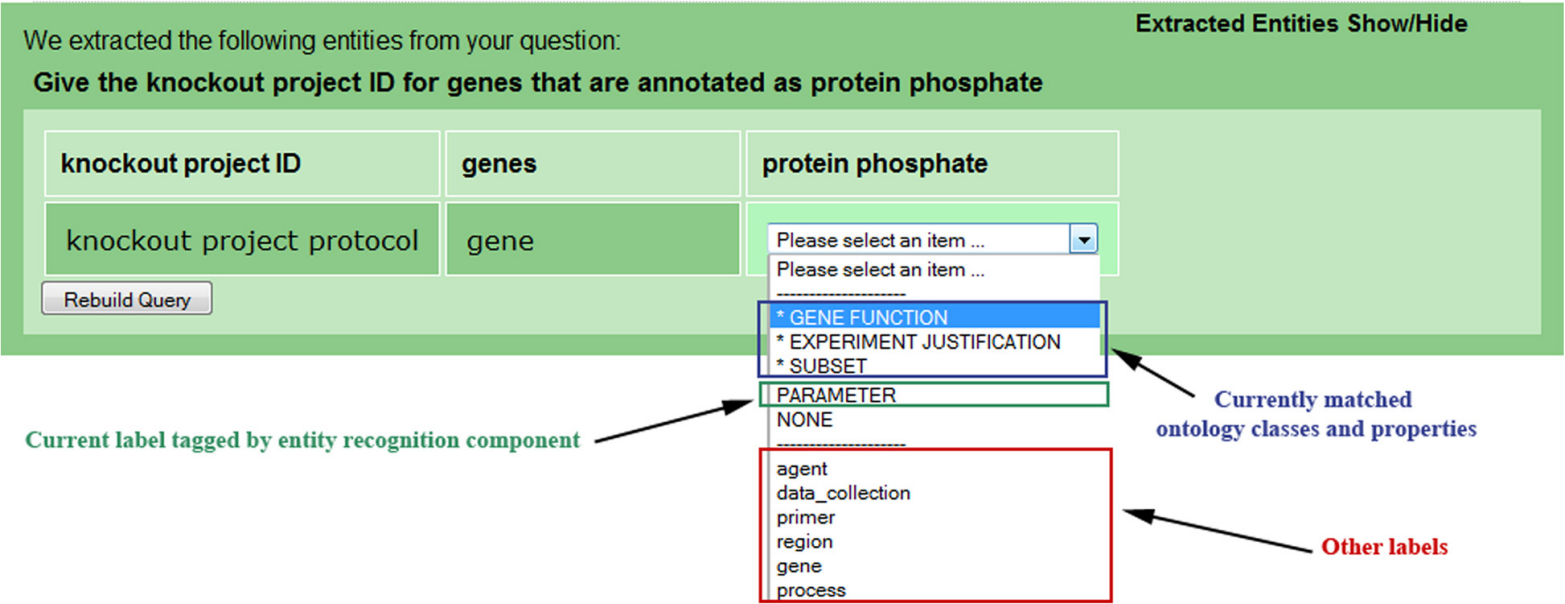
AskCuebee’s interface allows the scientist to revise the recognized entities and the labels comprising of matching ontology elements

Section (B) in Fig. 10 displays the output of three methods: *linguistic pre-processing*, *entity recognition* and *ontology element matching*. The processed question is displayed (in the green box) above the smaller (light green) boxes. Notice that the punctuation symbols are removed and comparative relationships are extracted and converted into a specific format that is readable for the system. For example, “above 1” is converted into either “greater than 1” or “ > 1”. In addition, section (B) displays the identified entities in the question and their corresponding labels, which are ontology classes and properties (in small light green and dark green boxes, respectively).

Importantly, AskCuebee allows the informed scientist to revise the identified entities and ontology-based labels in case the system has missed important entities or mislabeled an entity. Figure 11 focuses on section (B) for clarity. The text box containing *amastigote* entity turns gray when the user selects it and enables her to revise its content. In addition, clicking on the boxes containing the labels (dark green under the entity boxes), drops down a list with multiple options. This list shows all the lexically matching ontology classes and properties specially tagged by an asterisk, which is output from the *ontology element matching* component. The scientist may choose a different label or even remove an entity by selecting *NONE*. All candidate labels are listed below the horizontal line in the drop down list. This feature is significant because it allows the expert scientist to correct for any erroneous labeling. Finally, the scientist rebuilds the query using the *Rebuild Query* button.

In the next step, AskCuebee applies its unique *semantic association discovery* to the identified ontology elements. Consequently, the discovered RDF triples are displayed using Cuebee’s visual interface. These RDF triples are depicted in section (C) of Fig. 10. AskCuebee seamlessly utilizes the functionality of enhanced Cuebee from this point onwards, allowing the scientist to revise the sequences of triples if needed. The final component of AskCuebee, *query formulation and answer retrieval*, transforms these triples into a SPARQL query and retrieves the answer from the data sets. Section (D) of Fig. 10 shows the answer to the original question.

### Evaluation of AskCuebee

As we discussed in the previous section, multiple methods are available for realizing each component of OntoNLQA. We evaluate many of these methods in the *T. cruzi* context, and make an informed choice on the method that is finally used in AskCuebee.

The data sets and the lists of target questions utilized in our evaluation is described next followed by the evaluation methodology.

#### Data sources and target questions

**Fig. 12:**
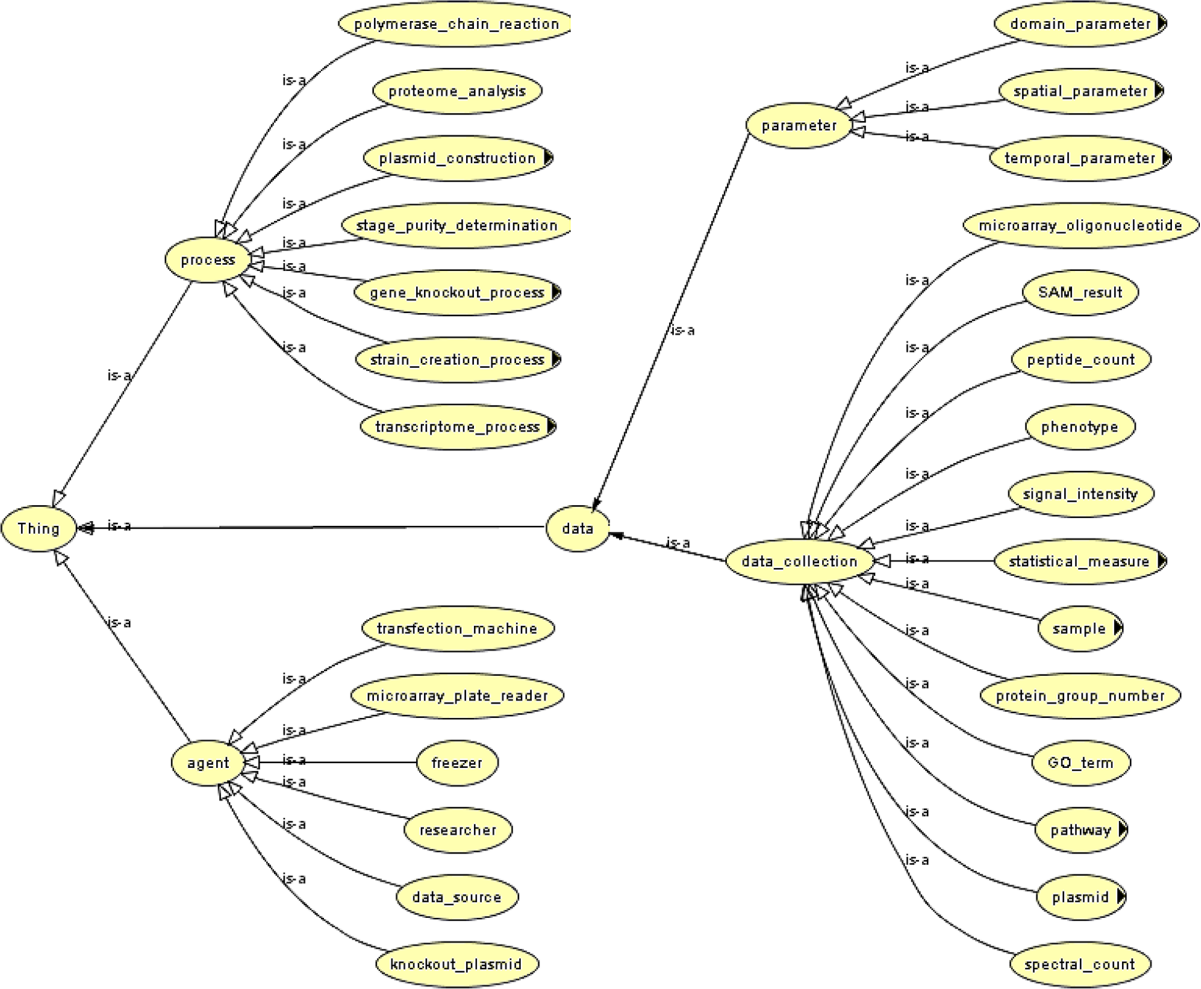
An excerpt of PEO limited to showing the class-subclass hierarchy. Object- and data-type properties and restrictions on classes and properties are not shown for clarity

AskCuebee forms the new question-answering interface for the semantic parasite knowledge repository [4]. Data in the repository utilizes the RDF data model and is annotated by two OWL ontologies: PEO and the Ontology for Parasite Lifecycle [66]. As we noted previously, PEO is a provenance ontology and models the experimentation processes used to generate parasite data, the description of raw materials, and the instruments and parameter values that influence generating or processing data. Figure 12 is a snapshot of PEO, which is available at NCBO’s BioPortal [67]. The other ontology describes the life-cycle stages of parasites, *T. cruzi*, *T. brucei*, and *Leishmania major*, including the host, parasitic and vector organisms, and anatomical location corresponding to each life-cycle stage.

Data sources accessed by AskCuebee include internal lab data and data sets from public repositories. We list the data sets below:

Internal lab data from the Tarleton Research Group at UGA:

- *Gene knockout*: data on DNA cloning steps required to generate gene knockout plasmids;
- *Strain creation*: data on creation of gene knockout strains in *T. cruzi* by transfection of parasites with gene knockout plasmids;
- *Microarray*: data on genome relative transcript abundances for the life-cycle stages of *T. cruzi*;
- *Proteome*: data on protein identification based on peptide spectra retrieved from *T. cruzi*’s life-cycle stages.

Data from public repositories, TriTrypDB and KEGG:

- TriTrypDB
  - *Orthologous genes* in organisms related to *T. cruzi* such as *T. brucei* and *L. major*.
  - *Predicated signal peptide information* from sequence based predictions for *T. cruzi* annotated gene regarding the likelihood of the gene product containing a single peptide;
  - *Transmembrane domain count* from sequence-based predictions for each *T. cruzi* annotated gene regarding the presence and number, if any, of transmembrane domain contained in the gene product.
- KEGG
  - *Pathway* data for *T. cruzi* genes regarding their presence or absence in a KEGG annotated metabolic pathway.

These data are transformed into the RDF data model and hosted in OpenLink Virtuoso 7, which is a fast RDF store. The ontologies are additionally loaded in a reasoner called Pellet [68], which allows queries to utilize the inferred information as well.

Several researchers from the Tarleton Research Group and other groups investigating *T. cruzi* together contributed two lists of 125 and 40 questions, respectively.^2^ These make an exhaustive set and are relevant to the day-to-day research activities of parasitologists investigating *T. cruzi* (see Additional files 1 and 2 for the lists of these questions). While the domain of these questions is limited to *T. cruzi*, they represent the type of common questions that researchers investigating other organisms may have. A shared characteristic between many of these questions is that they involve concepts and data that span over multiple data sources. For instance consider the question:

*What are the metabolic pathways related to protein group 271 for the orthologous genes with spectral score below 2.0?*

This question requires *gene knockout* and *proteome* internal lab data as well as *orthology* and *pathway information* from TriTrypDB and KEGG. Table 1 shows three example questions and the corresponding data sources providing the answers.

**Table 1:** Three example questions among a corpus of 125 questions for training and evaluating AskCuebee. The first column shows the question and the second column shows the data sets required to answer the question

Question	Data sources involved
Give the KO ID, gene ID, gene name, researcher notes, and knockout plasmid IDs for all genes that have orthologs in T.brucei and leishmania.	Gene knockout from TRG Ortholog information from TriTrypDB and KEGG
Find 5’ forward regions and all pathways for amastigote stages with log2 ratio above 1.	Microarray from TRG Gene knockout from TRG Pathway from KEGG
What are the metabolic pathways related to protein group 271 for the ortholog genes with spectral score less than 2.0?	Gene knockout from TRG Proteome data from TRG Strain creation data from TRG Ortholog information from TriTrypDB and KEGG Pathway data from KEGG

#### Methods for evaluation

##### Evaluating CRF for entity recognition

Identifying and labeling entities in the natural-language questions is a two-step method: In the first step, AskCuebee utilizes a CRF for identifying the entities and initially labeling them. In the second step, more specific labels are obtained by searching portions of an ontology. An efficient implementation of CRF exists in the Mallet package [69], which was utilized in AskCuebee. We use 8 initial labels in the training set obtained from the corpus of 125 questions relevant to *T. cruzi* immunology.

In order to evaluate the performance of the CRF, we perform 5-fold cross validation using the corpus of 125 questions. Each fold consists of 25 questions randomly selected from the corpus. We report the recall, which is the proportion of all entities that were correctly identified and labeled by the method, and the *precision*, which is the proportion of the identified entities whose labels are correct. In other words, the latter is a measure of the false positives. Correct classifications for the terms or phrases in each question in the corpus were independently identified by two parasitologists in the Tarleton Research Group and checked for agreement.

##### Evaluating string similarity measures for ontology element matching

Initial labeling by the CRF is followed by a dictionary-based look up method in AskCuebee. Instead of looking up each noun or verb phrase of the question in PEO or the data, CRF identifies the entities and provides an initial set of labels, which are the upper-level classes and properties in PEO. This helps by narrowing down the search for more specific labels to the portion of the ontology which has the initial label as the root instead of looking up the whole ontology.

In order to select a suitable string matching technique for use in AskCuebee, we evaluate all five text similarity measures.

Each of these measures provides a score between 0 and 1 which is considered as the degree of similarity between two sequences of characters.

This evaluation informs two decisions: The first is to identify the most appropriate similarity measure for our context. The second is to find the best threshold for the similarity score which would then be used to distinguish between correct and incorrect matches. Consequently, we evaluate the five similarity measures using five thresholds: 0.5, 0.6, 0.7, 0.8, and 0.9. While considering a higher threshold may result in more confident matches, we may fail to pick some of the possible matches; this is reflected in the *recall* metric. On the other hand, utilizing a low threshold may help us retain more possible matches but it increases the chances of obtaining incorrect matches, which is reflected in the *precision* metric. Consequently, we analyze the trade off that exists between finding more possible matches while minimizing the loss of precision. This trade off is minimized by examining the thresholds and selecting the measure and threshold which gives the highest *F1 score*.

The precision, recall and rejection rates [70] are measured as follows:

$$ \text{precision} = \frac{\text{number of correctly matched labels}}{\text{number of all matched labels}} $$

$$ \text{recall} = \frac{\text{number of correctly matched labels}}{\text{number of all correct labels}} $$

$$ \text{rejection} = \frac{\text{number of correct dissimilar matches}}{\text{number of all dissimilar matches}} $$

While precision and recall are commonly measured, the rejection rate is reported infrequently. It informs us about the false negatives – these are the labels which were deemed to be dissimilar matches but are correct – and is informative about the appropriate threshold.

In addition to matching with names of ontology classes and properties, the similarity measures are used for lexically matching the recognized entities with *rdfs:comment* and *rdfs:label* of ontology elements as well. This is especially important for biomedical ontologies where the class names are often identifiers with the descriptive information contained in the label or comment tags. If no lexical matches are identified in the ontology schema, we look up the RDF data in the parasite knowledge repository to find a match with instances (instance matching). As we explained previously in case (2) for the ontology element matching component of OntoNLQA, we rank multiple matches based on how many paths each has with other labels found in the ontology. The candidate with the most paths is retained.

For example, in the question:

*Find all genes with spectra score greater than 2.*

The phrase *spectra score* is identified as an entity and initially labeled by the CRF as DATA-COLLECTION. Subsequently, it is straightforwardly matched to ontology class, *spectral count*, using ISUB, which is within the portion of the ontology rooted at *data collection*. However, consider the question:

*Give the KO ID for genes that are annotated as protein phosphate.*

Note that acronym KO abbreviates knockout. As we mentioned previously, the interface allows some common acronyms in questions. The phrase, *protein phosphate*, is identified as an entity and labeled by the CRF as PARAMETER. It did not match closely with any ontology class or property under PARAMETER; instead appearing among the instances of *gene function*, which is a class under PARAMETER. Thus, in this case, AskCuebee employs instance matching to find the matching ontology class.

An alternative method for matching with instances utilized by previous approaches [21, 71] is to use *Apache’s Lucene* indexing [72] to search for the classes that include terms of the identified entities as their individuals. Lucene may speed up the ontology instance look-up process. However, Lucene does not access the RDF data contained in the RDF triple store. It requires direct access to the RDF files in order to index and search them. This requires maintaining another copy of the entire dataset, which we avoid in AskCuebee.

Previously, we discussed the method for evaluating lexical matching with ontology schema elements and instances separately. Next, we combine the two in order to evaluate the overall performance of this step. We analyze the precision, recall and F1 measure for matching identified entities with more specific ontology schema based labels.

##### Evaluating semantic association discovery

In order to find the most suitable *semantic association discovery* method for *T. cruzi* data, we evaluate the two approaches that OntoNLQA suggests from two perspectives: time and performance. The time evaluation demonstrates the average time that each approach takes to discover semantic associations for a set of ontology elements. The performance evaluation on the other hand, focuses on calculating the precision and recall metrics, which are computed here as shown below. The precision as calculated below is over all questions.

$${}{\small{\text{Precision} = \frac{\text{number of RDF triples generated that are correct}}{\text{number of all RDF triples that are generated}}}} $$

$${}{\fontsize{7.5}{6}{\text{Recall} = \frac{\text{number of questions for which RDF triples generated are correct}}{\text{total number of all questions that can be answered}}}} $$

##### Evaluating the full system

The performance of each component in the workflow of AskCuebee affects the performance of the full system. Therefore, we evaluate the performance of the system as a whole on our corpus of 125 questions using a 5-fold cross-validation and on a new corpus of 40 questions related to *T. cruzi* immunology not made available to the system previously in any way.

As AskCuebee allows user interventions during which the scientist may make simple refinements to the output of various methods including the RDF triple query in Cuebee (see Fig. 11), *four* scenarios present themselves:

1. Evaluation without any user refinements. This takes into account the errors of all the components;
2. User intervenes to fix errors in identifying entities. This evaluation takes into account any error from succeeding steps such as finding the specific labels for the entities and discovery of semantic associations;
3. User intervenes to correct errors in identifying entities and obtaining correct labels. This accounts for any error in semantic association discovery.
4. Finally, user intervenes to correct the output of all components including the final sequence of RDF triples. With no errors left uncorrected, AskCuebee offers its best performance.

A disadvantage of linked components in AskCuebee is that any error early on may propagate. For example, an error in identifying the correct entity and its label in the question below propagates throughout the system:

*Give the experimental notes for all KO genes in which their annotated function is protein phosphate.*

Let us assume that the CRF identifies the entity, *annotated function*, with the label DATA-COLLECTION instead of PARAMETER. This error is passed on to ontology element matching where the incorrect subclass from PEO, *peptide count*, instead of *gene function* is matched with the entity. Consequently, an incorrect set of ontology elements are used for discovering semantic associations leading to an incorrect RDF triple query.

For this evaluation, similar to others, we start with the corpus of 125 questions. An evaluation of the full system focuses on the correctness of the answers obtained to the questions. While 88 of the 125 questions may be answered based on the data in our repository, the remaining 37 questions do not have any answer in our data. Therefore, for these we compute precision and recall by utilizing the correctness of the generated RDF triple query as the reference standard. AskCuebee (and specifically the CRF) is trained on 4 folds and tested on the fifth, with each fold randomly containing 25 questions. We repeat this process five times by rotating over the folds, to evaluate the system against all 125 questions.

We calculate the precision as the proportion of the questions that generate correct answers or queries among the number of questions that generate some answer or query (correct or incorrect). The recall on the other hand is the proportion of questions that produce correct answers or queries for the questions. Two parasitologists in the Tarleton Research Group independently identified correct records in the answers shown in tabular format for each question. Usually, certain records are expected to be present in the answer of each question, and often all such records were present or all were absent. Evaluating the entire system based on the four scenarios provides us with valuable insight on how much error from the different components is propagated throughout the system.

##### Evaluation of full system on unseen questions

We evaluated the performance of AskCuebee on a corpus of 40 new questions not seen previously (see Additional files 1 and 2 for these questions). Similar to the previous evaluation, we calculate the precision and recall based on the correctness of answers (if they exist in the data) or correctness of the generated RDF triple query.

## Results and discussion

AskCuebee provides a context within which to implement OntoNLQA and evaluate the various methods for realizing each component of the framework. Overall performance of AskCuebee is also evaluated, which provides an indication of the utility of the framework.

### Results from component evaluations

CRF-based entity recognition in AskCuebee obtains an average precision of 93.29 % and an average recall of 91.35 %, with the F1 measure of 92.28 % across all the folds. The standard deviation across the folds for precision is 0.0219, for recall is 0.02523, and for F1 is 0.0170.

In order to evaluate ontology element matching, we first find the correct labels for the entities identified by the CRF in each question in our corpus. While 149 entities were identified, 102 of these had lexical matches with the classes or properties in PEO.

Tables 2, 3, 4, 5, and 6 demonstrate the results of our evaluation for different thresholds. In each table, the highest scores are marked in bold. The highest recall and F1 score is demonstrated by ISUB for the thresholds 0.5, 0.6, 0.7, and 0.8 though the corresponding precision is not the best. For the threshold of 0.9 however, Smith-Waterman based similarity produces higher scores than ISUB. As the results in Table 3 suggest, ISUB similarity has the highest F1 score (81.69 %) across all of the thresholds (marked with an asterisk). Therefore, ISUB with a threshold of 0.6 is selected for AskCuebee’s ontology element matching.

**Table 2:** Evaluating various similarity measures with a threshold of 0.5

Similarity measure	Precision	Recall	F1	Rejection
ISUB	81.69	79.45	80.56	99.30
Levenshtein	87.04	64.38	74.02	98.88
SmithWaterman	70.31	61.64	65.69	99.31
Cosine	89.80	60.27	72.13	98.83
Jaccard	90.24	50.68	64.91	98.45

**Table 3:** Result of similarity measure evaluation for a threshold of 0.6. The highest F1 measure appears for ISUB at this threshold

Similarity measure	Precision	Recall	F1	Rejection
ISUB	84.06	79.45	*81.69	99.34
Levenshtein	94.87	50.68	66.07	98.46
SmithWaterman	73.77	61.64	67.16	99.18
Cosine	90.24	50.68	64.91	98.45
Jaccard	92.31	32.88	48.48	97.90

**Table 4:** Result of similarity measure evaluation for a threshold of 0.7

Similarity measure	Precision	Recall	F1	Rejection
ISUB	86.15	76.71	81.16	99.31
Levenshtein	93.55	39.73	55.77	98.12
SmithWaterman	74.58	60.27	66.67	98.97
Cosine	91.18	42.47	57.94	98.20
Jaccard	94.44	23.29	37.36	97.57

**Table 5:** Result of similarity measure evaluation for a threshold of 0.8

Similarity measure	Precision	Recall	F1	Rejection
ISUB	89.47	69.86	78.46	99.01
Levenshtein	92.59	34.25	50.00	97.95
SmithWaterman	75.00	57.53	65.12	98.79
Cosine	92.31	32.88	48.48	97.86
Jaccard	92.31	16.44	27.91	97.36

**Table 6:** Results of similarity measure evaluation for a threshold of 0.9

Similarity measure	Precision	Recall	F1	Rejection
ISUB	96.88	42.47	59.05	98.16
Levenshtein	94.44	23.29	37.36	97.57
SmithWaterman	74.07	54.79	62.99	98.58
Cosine	100.00	13.70	24.10	97.28
Jaccard	100.00	15.07	26.19	97.32

Among the total of 149 entities identified in our corpus of 125 questions, 47 do not match with elements in the ontology schema; rather they are matched with instances using exact lexical matching provided by Virtuoso SPARQL queries. Here, instance matching displayed a precision of 78.37 %, recall of 70.73 % and a combined F1 score of 74.36 %.

Next, we present the results of combining the lexical matching with ontology elements and instances and show the overall performance of this step. Similarly to previous results, we report the precision, recall and F1 measure for matching the 149 identified entities with more specific ontology schema based labels. As we show in Table 7, ISUB-based matching with both ontology classes and properties, and instances significantly improves the performance to an F1 measure of 79.09 % compared with 63.39 % when just the classes and properties are matched, and 38.41 % when just the instances are matched. As we may expect, this increase is due to a significant improvement in the recall.

**Table 7:** Evaluating lexical matching of entities with ontology schema based elements and instances. Notice the improved recall when both are performed. We used ISUB for measuring the similarity

Approach	Precision	Recall	F1	
Combined matching with ontology schema and instances	82.08	76.32	79.09	
Matching with ontology schema only	84.06	50.88	63.39	
Matching with instances (RDF data) only	78.38	25.44	38.41	

As previous systems predominantly look up the identified entities in the RDF data to form the triples, we analyzed our corpus for those questions whose identified entities formed a triple that could be located in the data. We found 10 such questions indicating that less than 10 % of the questions may be answered in this simple way.

AskCuebee precomputes the pairwise LCAs for all classes in PEO using a fast algorithm. As the algorithm requires the graph to be acyclic while ontology graphs could be cyclic when named properties are included (see Appendix Appendix B), we first break any cycles in PEO’s ontology graph by introducing new nodes using the technique described previously in OntoNLQA. This increases the nodes of the graph from 144 (ontology classes) to 1,386. Transforming the cyclic graph and precomputing the all pair-wise LCA consumes 15.21 seconds on a high-end server having a six-core Xeon 2GHz CPU with 32GB of RAM. Given the precomputed LCAs stored in a look-up table, we obtain a single LCA between all labeled entities of a question and find the shortest paths from the LCA to the ontology classes. For all 125 questions in our corpus, the time consumed in obtaining the sequences of RDF triples given the LCAs was 126.85 seconds. We sum the two times and obtain the average time taken per question, which is 1.14 seconds. Note that the offline LCA computation is amortized over the questions, and it’s impact on the time consumed reduces as more questions are asked. A drawback of precomputing pair-wise LCAs offline is that if the ontology schema changes, the pairwise LCAs may change as well and would need to be precomputed again. When we regenerate the acyclic ontology graph, we emphasize the relationships that exist in the RDF data in the form of subject, predicate and object as these have instances. Therefore, even changes in the datasets requires recomputing pair-wise LCA.

For the alternative approach, we use AskCuebee’s RDF store Virtuoso’s query endpoint for path queries. All paths between each pair of ontology-based labels are found and their intersection provides the set containing the LCA. Then, analogously to the previous approach, the shortest paths are obtained from the LCA to the labels. The difference from the previous approach is that no offline precomputation is involved. Obtaining semantic associations between entity labels in this way consumes an average of 3.14 seconds per question in our corpus, with a large proportion of the time consumed by path querying. Clearly, the first approach is more efficient and is subsequently utilized in AskCuebee.

Table 8 gives the results of evaluating the correctness of the two approaches using precision, recall and a combination of the two. In addition to being the quicker of the two, precomputing the pairwise LCAs results in significantly better correctness performance.

**Table 8:** Results evaluating different approaches for semantic association discovery. The F1 score shows the significantly higher performance of LCA approach (90.80 %) compared to the alternative approach (77.6 %)

Approach	Precision	Recall	F1
LCA	91.86	89.77	90.80
Alternative	81.61	73.96	77.60

In conclusion, the precomputed LCA approach for semantic path discovery improves over the alternative approach in both time efficiency and accuracy in the context of *T. cruzi*. The F1 score for LCA approach is 90.80 % which is significantly higher than the alternative approach with 77.6 % score. The higher difference between the recall scores of 89.77 % for LCA compared to 73.96 % for the alternative approach provides evidence that the alternative approach suffers in discovering the RDF triples. Additionally, precomputing LCA is almost 3 times faster than the online approach.

### Results from full system evaluation

Table 9 summarizes the precision, recall and F1 measures of evaluating the full system in the four scenarios described in the previous section. These scenarios pertain to differing user interventions, which include editing the entities recognized by the CRF, selecting other labels for the recognized entities that replace those automatically found, and editing any component of the RDF triple query itself.

**Table 9:** Results of evaluating the full system in four scenarios. The last scenario is expected to represent the best performance of AskCuebee while scenarios 1, 2, and 3 include possible errors from different components of the system

Scenario	Precision	Recall	F1
Scenario 1	69.35	68.8	69.07
Scenario 2	72.58	72	72.28
Scenario 3	93.54	92.8	93.17
Scenario 4	97.58	96.8	97.18

A difference in F1 measure of 24.1 % between scenarios 1 and 3 indicates that correctly identifying entities in questions and matching these with labels in the ontology schema plays a critical role in improving the system performance. Notice that errors due to incorrectly identifying entities by the CRF contributes just 3.21 % to this difference. This motivates a focus on the lexical matching component. Errors in semantic association discovery have an impact on the correctness of answers with performance improving by 4.01 % due to correcting for such errors. This occupies 28.11 % of the overall improvement in performance due to user interventions with corrections of matched labels contributing the most. AskCuebee correctly answers 85 of the 125 questions without any user intervention.

#### Results on unseen questions

Table 10 presents the results of an evaluation of the full system on the unseen corpus of 40 questions. AskCuebee automatically answers 24 questions without any user intervention with the number increasing to 33 questions when the scientist corrects for any entity recognition and ontology element matching errors.

**Table 10:** Evaluation of AskCuebee on the corpus of 40 questions. These questions are not used in training any part of the system

Scenario	Precision	Recall	F1
Scenario 1	61.54	60	60.76
Scenario 2	64.10	62.50	63.29
Scenario 3	84.62	82.50	83.54
Scenario 4	100	97.50	98.73

### Discussion and limitations

AskCuebee does not limit the questions to a specific set or templates. Subsequently, the preprocessing does not match the question to a template. However, its use of dependency grammar makes it sensitive to the grammar of the question. Therefore, questions exhibiting the correct grammar are more likely to produce correct answers. Machine learning based entity recognition typically requires a large corpus of training data for reasonable performance. AskCuebee’s focus on a single organism confines the number of possible types of questions that are asked. We partially address this issue by combining machine learning based entity recognition to obtain abstract labels with lexical ontology look up for specificity. Of course, ontology classes and properties may not always provide a match with the recognized entities, in which case the framework suggests searching the annotated data for a match. Classes that annotate matched data serve as more specific labels.

Finding the LCA as a common point between the identified entities is a general way of obtaining the semantic associations between the entities given a transformed ontology graph. However, we identify four types of questions for which this approach gives incorrect associations: questions with negative expressions, questions with complex comparative relationships, questions containing one ontology entity only, and questions that use complex query patterns such as nested queries and queries on groups. As examples of such types, consider the following three questions:

1. *Find all gene knockout targets in amastigote stage that have orthologs in Leishmania but not in T. brucei.*
2. *Give the strain summaries for all amastigote genes that have a standard deviation less than 1.5 of the log2 ratio.*
3. *Show proteins that are downregulated in the epimastigote stage and exist in a single metabolic pathway.*

Question (1) contains a negation, which is lost in the association discovery step. Though the interface of Cuebee allows users to formulate such questions, AskCuebee is unable to obtain the correct query. Question (2) contains a relationship that involves comparing with a function of the values of two classes. While AskCuebee does not support such complex comparisons, we may address such comparisons by considering additional grammar dependencies. However, creating general rules is difficult because there may be many operations that could be considered.

In question (2), the user is interested in all the available data on only one entity, *cloned samples*. Cuebee requires at least two entities in order to generate an RDF triple with an object or data type property between them.

Finally, to formulate question (3) we require utilizing nested queries, group-by and aggregated functions. In particular, to answer this question we require a query that uses *Group by* to group all the epimastigote genes associated with a single metabolic pathway (group by genes that have a pathway count of 1).

Intervening to correct the RDF triple query or enter a new RDF query can generate answers for some questions that involve negation and group-by clauses. This is because the triple query interface Cuebee supports these operations. In the previously unseen corpus of 40 questions, 6 had negations in them while 1 pertained to a single concept.

## Related works

Several systems allow querying of semantic data annotated using domain ontologies. We may categorize these into two groups based on how the query is posed: (*i*) approaches in this group solicit queries as RDF triples, or ask users to select points on a graph, or use other visual representations. In these systems, the scientist must transform the question in her mind to the representation sought by the system; (*ii*) these approaches solicit questions in their natural form, and minimal, if any, transformation of the question is needed.

### Query answering systems

Specific to biomedicine, BioSPARQL [10] facilitates querying biomedical linked open data. It finds the concepts in the ontology that are lexically most similar to a phrase provided by the user. A subgraph with the selected concept in the center and surrounded by related concepts is formed. The user may select another concept in the subgraph and BioSPARQL finds all paths between the two concepts with a facility to create a more specific query. BioGateway [73] composes several online ontologies from OBO foundry [74], GO annotation files [75] and in-house data sources, and provides a single entry point (gateway) to query using SPARQL. Pre-formulated SPARQL queries are available and may be refined. Analogously, Cheung et al. [76] provide facilities to integrate different semantic data stores in neuroscience, and offer either SPARQL or SQL query interfaces to access the remote data.

iSPARQL [8] allows users to select concepts and relationships in an ontology and connect them using the provided graphical tools.

NITELIGHT [9] extends iSPARQL by providing a query design canvas that allows the user to move the elements around and edit them through menu items.

Hogenboom et al. [77] also offer a visual interface using a SPARQL-based graphical query language for RDF called RDF-GL. It uses boxes to represent ontology classes and arrows for the properties between them. Hogenboom et al. also use cycles to depict different operations such as union or Boolean operations for data types. Knowledge about SPARQL is needed in order to use many of these systems.

### Natural language question answering systems

Systems in this category accept queries expressed as natural language questions, and utilize ontologies in the process of analyzing the question and returning answers from RDF data stores that subscribe to the ontology. Therefore, the scientist need not learn the vocabulary or structure of the underlying ontology, unlike the systems discussed in the previous subsection. Lopez et al. [78] provide a survey of semantic question answering systems.

Closely related to AskCuebee is AquaLog [19] which utilizes the well-known general architecture for text engineering (GATE) [79] and the Java annotations patterns engine (JAPE) to tokenize and tag phrases in the question with parts of speech annotations.

These annotations are used to create linguistic triples each consisting of the subject, relationship and object. AquaLog then maps the triples to concepts and properties in the ontology using lexical matching assisted by WordNet. AquaLog is evaluated on two distinct data sets [80, 81] using 69 and 68 questions, respectively, and reported a 58 % and 69.11 % success rate in answering the questions (without user intervention).

A recent extension of AquaLog [82] provides support for multiple ontologies and larger data. OntoNLQA’s use of the LCA to form queries that make intuitive sense distinguishes it from AquaLog, which directly forms a group of triples from the question, and contributes to a better precision.

LifeQA [83] proposes to build SPARQL queries from natural language questions in the context of the life sciences. Based on an analysis of 14 questions from the 2007 TREC genomics track [84], Kim et al. suggest a 5-step process: named entity recognition, parsing, targeting, conditioning, and encoding. In agreement with OntoNLQA, Kim et al. suggest that finding shortest paths between important entities detected from previous steps is sufficient to encode a SPARQL query for the original question. However, no approach is proposed and no prototype system exists to the best of our knowledge.

Guided-input natural language question-answering, GINSENG [11], relies on a simple question grammar, which is extended using the ontology schema to guide users to directly formulate SPARQL queries. Evaluation on multiple datasets, not in the biomedical domain, demonstrates that the system achieves very high precision of 97 % and more due to its direct use of the ontology in posing the question, but poor recall of 40 % or less. SemanticQA [21] shares its approach with GINSENG by providing a facility to assist users in constructing their question as they type. It presents valid suggestions in the universe of discourse of the selected ontology, whose content has been previously indexed with Lucene [72]. Linguistic triples are extracted from the question and searched in the RDF data; those that are incomplete are referred to Web documents (e.g., PubMed) to complete them. A small scale ad-hoc test performed with only 8 samples of simple factoid questions using the Lehigh University Benchmark ontology yielded 63 % precision, and 6 sample queries using the SwetoDBLP ontology yielded 83 % precision. Neither GINSENG nor SemanticQA perform path finding instead simply forming a group of RDF triples, which constitute the SPARQL query.

NLP-Reduce [20] treats natural language questions as a bag of words and differs by making reduced use of linguistic processing; it utilizes just stemming. NLP-Reduce matches the bag of words from a parsed question to the synonym-enhanced triples stored in a lexicon and generates SPARQL statements for those matches. All the triples for which at least one of the question words occurs as an object, property or literal in the triple are retrieved, and it seeks to form a chain of triples which covers the entire question. This approach differs from OntoNLQA’s approach of finding the LCA in order to form a sequence of RDF triples. Of course, not all relationships are explicitly stated in a question. Consequently, NLP-Reduce often finds it difficult to form the correct chain. Kaufmann et al. [85] evaluated NLP-Reduce on the same non-biomedical datasets as GINSENG leading to a high precision and improved recall.

Another system in this category is FREyA (feedback, refinement and extended vocabulary aggregation) [86], which is a knowledge-based question answering system that incorporates ontology reasoning and syntactic parsing. Questions are parsed to produce parse trees using GATE and OntoRoot Gazetteer [79]. Nodes in the parse trees are matched with ontology elements and generation of the final SPARQL query solely depends on the matched ontology concepts rather than a semantic association between the concepts. Therefore, the system fails to generate an answer if a required property is not mentioned in the original question. Nevertheless, an evaluation on 250 geographical questions resulted in a precision and recall of 92.4 %. As we mentioned previously, less than 10 % of the questions in our corpus exhibit triples that are directly found in the data.

## Conclusion

In this article, we introduced a new framework for ontology-based question answering. OntoNLQA offers five steps in order to answer natural language questions. OntoNLQA may be utilized for any domain that is supported by one or more ontologies. As we show in this article, selection of specific methods to realize the component approaches involves evaluating them in the context of the specific ontologies and RDF data that is available.

The primary contribution of OntoNLQA is its capability of modeling the semantics of the question and mapping them to the semantics of the annotated data model (ontology and RDF triples), in order to generate computational queries for retrieving the answers. Moreover, in designing the framework, we are cognizant of the fact that the scientist may have no prior knowledge about the underlying ontology’s design and its structure. As a result, another contribution of our approach is its hiding of the complexity of the ontology schema and the query language from the scientist.

We applied OntoNLQA in the context of *T. cruzi* parasite research, resulting in AskCuebee. We evaluated various alternative methods in order to realize each step in AskCuebee. Additionally, we performed a comprehensive evaluation of the system as a whole and discussed the effect of errors in each component on the overall performance of the system. In comparison to previous systems, AskCuebee differs in how some of the methods are realized. Main conclusions from these evaluations are listed below:

1. Lexical similarity measure, ISUB, demonstrates matching performance better than many other measures, making it well suited for use in ontology element matching.
2. Precomputing all-pairs LCA is significantly quicker and improves correctness compared to the online path query approach.
3. Matching recognized entities from questions with ontology classes, properties and data for specificity significantly improves performance of the system.
4. Automatically recognizing key entities in questions followed by identifying related concepts in ontologies and finding the associations between them using LCA allows answering 68 % of the questions, which indicates that this approach performs well.
5. Through these evaluations and an understanding of the methods, we identified four types of questions, which may not be answered by AskCuebee.

We have prioritized supporting questions with negations and aggregate operators as the next step in future work.

## Appendix A

### Background on CRF

**Fig. 13:**
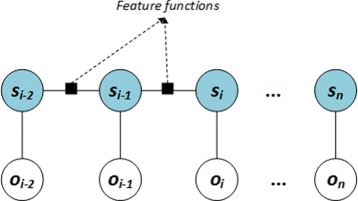
The graphical representation of linear-chain CRFs containing feature functions

CRFs are undirected statistical graphical models that compute the conditional probability of values on output nodes given values assigned to input nodes. In special cases, the input nodes of the model are linked by edges in a linear chain under the first-order Markov assumption such that the distribution over a node is conditioned on the value of the previous node only and not the entire history. We may view these linear-chain CRFs as conditionally trained finite state machines. Figure 13 illustrates the graphical representation of CRFs.

Let *o*=〈*o*_1_,*o*_2_,...,*o*_*n*_〉 be a sequence of observed input data of length *n*, such as a sequence of words in a sentence. Let *S* be a set of finite state machine states with corresponding labels, *l*∈*L*. Examples of labels in our context include *process*, *data collection* and *agent*, which are the higher-level classes in the hierarchy of PEO. Let *s*=〈*s*_1_,*s*_2_,...,*s*_*n*_〉 be the sequence of states in *S* that correspond to the labels assigned to words in the input sequence, *o*. The linear-chain CRF defines the conditional probability of a state sequence given an input sequence as:

(1) $$ P(s|o) = \frac{1}{Z_{o}} exp \left(\sum_{i=1}^{n} \sum_{j=1}^{m} \lambda_{j} f_{j}(s_{i}, s_{i+1}, o, i) \right)   $$

where *Z*_*o*_ is a normalization factor over all the state sequences, *f*_*j*_(*s*_*i*−1_,*s*_*i*_,*o*,*i*) is a function that describes features (see Fig. 13), *m* is the total number of feature functions, and *λ*_*j*_ is the learned weight for each of the feature functions. A feature function may be defined to have value 0 in most cases and value 1 in other cases. For example, Table 11 gives a list of the orthographic features used in training the CRF.

**Table 11:** List of orthographic features used in training the CRFs model

Orthographic feature	Regular expression
HASDASH	.*-.*
INITDASH	-.*
ENDDASH	.*-
INITCAPS	[A-Z].*
INITCAPSALPHA	[A-Z][a-z].*
REALNUMBERS	[-0-9]+[.,]+[0-9.,]+
NATURALNUMBER	[0-9]+
ALLCAPS	[A-Z]+
CAPSMIX	[A-Za-z]+
DIGIT	.*[0-9].*
SINGLEDIGIT	[0-9]
DOUBLEDIGIT	[0-9][0-9]
GENEPATT	.*[tbglmjfrnix0-9]+[.][0-9]+.*
DNASEQUENCE	[ACTG]+
HASROMAN	.*\\b[IVXDLCM]+\\b.
ROMAN	[IVXDLCM]+

## Appendix B

### An efficient algorithm for computing LCA

Baumgart et al. [87] proposed an efficient method for finding LCAs in a directed acyclic graph. Other methods such as using a range-minimum query [88] also exist. Baumgart’s algorithm utilizes the concept of shortest ancestral distance between two nodes in a graph. For every node pair, *x* and *y*, the algorithm finds a list of all nodes, *C*, where for every *c*∈*C*, sum of *distance*(*c*,*x*) and *distance*(*c*,*y*) is minimal. This method is the most efficient so far to the best of our knowledge.

**Fig. 14:**
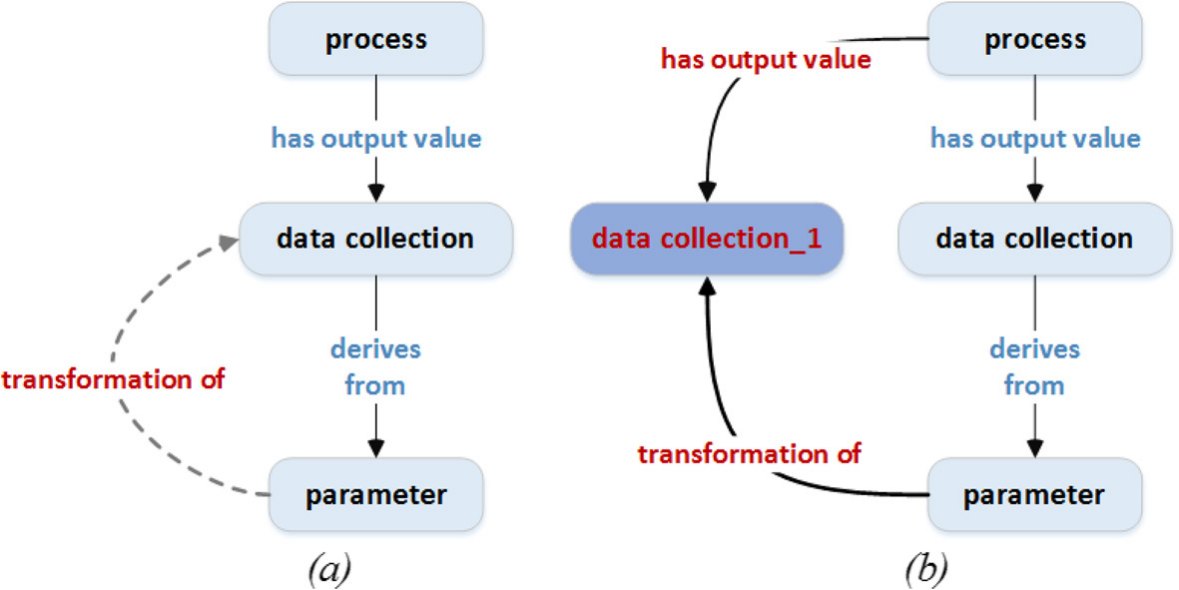
We avoid cycles in the graph by splitting a node. In the subgraph (**a**) an edge, *transformation of*, between two nodes, *parameter* and *data collection*, causes a cycle. Subgraph (**b**) shows the edge, *transformation of*, is added between *parameter* and a new node with a similar name, *data collection_1*

A complication is that Baumgart et al.’s algorithm works on directed acyclic graphs only while ontologies are often cyclic graphs when the named properties are considered. Therefore, we must convert the ontology graph into a directed acyclic graph with no loss of information. The framework transforms a cyclic graph into a acyclic one while retaining the information from the cyclic graph. The graph is regenerated; a cycle is detected by remembering the previously generated nodes, and the repeated node that causes the cycle is split into two nodes with no edge between them (see Fig. 14 for illustration). A naming convention for the newly created nodes is utilized to remember that these split nodes constitute a single particular node in the original ontology. We must remember these nodes to avoid creating excess ones. Specifically, a node deemed to be repeated is checked if it has been split previously. If so, then the edge may be added to the split node if no cycle results. Note that multiple LCAs may exist between two classes and each of these is computed.

The LCA algorithm builds a table of *n*×*n* dimensions where *n* is the number of classes in the ontology. Each cell of the table contains all possible LCAs between the corresponding ontology class pair. If we index the class pairs, we may retrieve the LCAs in near-constant time. The table would be regenerated when there is a change in the ontology schema.

## Endnotes

^1^AskCuebee is available for use at http://jade.cs.uga.edu.

^2^ Note that no publicly available corpus of questions on parasite research exists in our knowledge.

## Additional files

Additional file 1
**Question list 1.** This file contains the corpus of 125 questions related to parasite immunology research. It is utilized for the five-fold cross validation.

Additional file 2
**Question list 2.** This file contains the corpus of 40 questions related to parasite immunology previously unseen by the system. It is utilized in the evaluation of the system.
